# Delayed Postural Responses to Fear of Falling During Gait Initiation in Low Vision: Insights from Virtual-Reality-Based Fear Simulation

**DOI:** 10.3390/healthcare14030400

**Published:** 2026-02-05

**Authors:** Mansoo Ko, Praveena K. Gupta, Gregory Brusola, Metha R. Chea, Pranati Ahuja, Tony Chao, Rodney L. Welsh

**Affiliations:** 1 Department of Physical Therapy, School of Health Professions, University of Texas Medical Branch at Galveston, Galveston, TX 77555-1144, USA; gabrusol@utmb.edu (G.B.); tochao@utmb.edu (T.C.); rlwelsh@utmb.edu (R.L.W.); 2 Department of Ophthalmology and Visual Sciences, School of Medicine, University of Texas Medical Branch at Galveston, Galveston, TX 77555-1144, USA; prgupta@utmb.edu (P.K.G.); mrchea@utmb.edu (M.R.C.);

**Keywords:** fear of falling, gait initiation, virtual reality, low vision, postural control

## Abstract

**Purpose:** This study aimed to quantify the severity of fear of falling (FOF) in people with low vision (LV) compared with age–gender-matched healthy individuals during gait initiation (GI). **Methods:** A total of 14 adults with LV and 14 age–gender-matched healthy adults were recruited from local communities. The Falls Efficacy Scale International was used to evaluate FOF. We compared temporal events between healthy and LV groups. For the healthy group, GI under normal vision was further compared to conditions using a low-vision sight simulator (SS) and an immersive virtual reality (VR) environment designed to simulate a fear-evoking experience. Independent *t*-test and one-way repeated measure ANOVA were conducted for statistical analysis (*p* < 0.05). **Results:** People with LV showed a significantly greater FOF than healthy individuals (*p* < 0.05). During GI, participants with LV exhibited significantly prolonged anticipatory postural adjustment (APA) durations compared to healthy normal and SS conditions (*p* < 0.05). While VR-evoked fear in healthy participants primarily prolonged the push-off (PO) phase, the delay in the LV group was characterized by a significantly extended initial anticipation (AP) phase. Notably, the APA duration in the LV group showed no significant difference compared to the healthy VR condition, indicating that the inherent fear in LV produces postural delays as severe as those induced by extreme VR-evoked fear of heights (*p* > 0.05). **Conclusions:** This study demonstrates that individuals with LV adopt a chronically conservative motor program during the transition from standing to walking. These postural hesitations are statistically comparable to those observed under fear-evoking, VR-induced environments. These findings suggest that LV is associated with a distinct biomechanical strategy that prioritizes static stability over dynamic movement. Accordingly, multidisciplinary rehabilitation approaches that emphasize sensory reweighting, including vestibular training, alongside interventions targeting FOF, may be essential for mitigating altered postural control and reducing fall risk in the LV population.

## 1. Introduction

Visual impairment (VI) is a primary cause of loss of independence among older adults. Globally, over 2.2 billion people live with VI, resulting in substantial annual costs for long-term services [1]. The total annual economic impact of vision loss and blindness in the United States reaches approximately $134.2 billion [2]. Specifically, fear of falling (FOF) is a major health concern for older adults with VI, often leading to complications such as falls, hip fractures, anxiety, depression, immobility, and functional dependence [3,4]. An estimated 48.3% of older adults with VI report FOF due to poor visual acuity, reduced contrast sensitivity, and loss of depth perception [5,6]. This fear significantly impacts anticipatory postural control, the process by which individuals adjust their postural balance or prepare to move prior to executing the first step from a standing position. Consequently, this FOF often triggers protective strategies such as stiffening the body, hesitating before initiating movement, or delaying actions, serving as preventive measures against potential falls [7]. Of equal concern is the contribution of FOF to fear-related activity and participation restriction in people with VI, with significant implications on their overall functional mobility [1,5,8].

Gait initiation (GI) is defined as a transitional period between quiet standing and the initiation of steady state walking. This common motor task is performed throughout the day by ambulatory individuals and is used to assess balance control during gait [9]. In regular walking patterns, GI includes both double and single support phases. It involves anticipatory postural adjustment (APA) to counteract the destabilizing postural balance caused by a rapid backward weight shift. Individuals with balance deficits often show reluctance to disrupt their static balance by delaying weight shifting during GI [9]. This delay in GI has been associated with the increased risk of multiple falls [10] and has recently been found to be strongly associated with increased reliance on vision in older adults [11]. Accordingly, the task of GI may maximize FOF-related control strategies to execute the first step by breaking the static balance in older adults with low vision (LV). Vision is critical for planning goal-directed movements because it defines the kinematic plan and modulates the scaling of movement prior to movement initiation [11,12]. Previous studies have shown that postural threat, such as exposure to elevated surfaces or virtual height environments, attenuates anticipatory postural adjustments and constrains weight shifting during gait initiation (GI) in healthy adults [13]. However, it remains unclear how fear disrupts movement control strategies during GI in individuals with LV. To address this gap, experimental paradigms that can systematically manipulate fear-related contextual factors during gait are essential. Traditional approaches to simulate visual impairment include optical blur lenses and prism-based visual perturbations [14,15]; however, these methods primarily alter sensory input and are limited in their ability to dynamically manipulate environmental threat and fear-related contextual factors during walking tasks [16]. Compounding these sensory constraints, conventional experimental paradigms frequently rely on highly repetitive tasks, which can reduce participant engagement and compromise sustained attention, particularly in frail older adults and clinical population. To address these limitations, virtual reality (VR)-based locomotion paradigms incorporate interactive and goal-directed tasks, such as collecting virtual targets via a full-body embodied avatar, to enhance motivation and mitigate the inherent monotony of gait-related protocols [17]. Beyond improving engagement, this approach enables the extraction of sensitive, trajectory-based metrics, including Normalized Path Length and Initial Angle Error, which provide quantitative measures of visuomotor adaptation and motor control performance [17]. Such high-fidelity data collection is particularly valuable in clinical settings for establishing recovery benchmarks and tailoring interventions for populations with neuromotor disorders.

Given that FOF is a multifactorial construct bridging physical instability and psychological apprehension, VR offers the unique advantage of systematically isolating and manipulating fear-related variables within a controlled, yet safe, environment [18,19]. VR-based technology allows for the simulation of visual input related to poor visual acuity, reduced contrast sensitivity, loss of depth perception, and vertigo, leading to FOF. Importantly, simulating visual impairment in healthy individuals facilitates the acquisition of larger, more standardized datasets than are typically feasible with visually impaired populations. This approach is particularly valuable because data collection from individuals with actual visual or neuromotor impairments is often constrained by substantial practical and safety-related limitations. Due to physical fatigue, balance instability, and fall risk, pathological populations typically tolerate only a limited number of task repetitions and require longer rest periods between trials. Sample sizes are therefore frequently restricted, particularly in individuals with non-autonomous gait execution, and experimental sessions commonly necessitate continuous physician or clinical staff supervision to ensure participant safety. These constraints can substantially limit the volume, variability, and standardization of gait data that can be acquired from clinical populations. Recent work has highlighted these challenges and emphasized that simulating pathological gait or sensory impairments in healthy participants, when conducted under appropriate clinical guidance, provides a principled methodological strategy for acquiring larger and more standardized datasets prior to validation in clinical populations [20]. By enabling a greater number of repetitions under controlled conditions, this approach directly addresses the structural limitations inherent to clinical data acquisition and establishes a clear methodological rationale for the proposed workflow, while maintaining the safety and feasibility required for clinical research.

Consequently, this methodological framework mitigates common experimental constraints, such as limited task repetition and the extended recovery periods required by clinical participants due to fatigue and safety considerations. Previous studies have attempted to simulate VI in healthy individuals [21,22,23], but limited research exists on simulating and assessing FOF. It is still unknown whether the anxiety perceptions experienced in real environments mirror those in virtual contexts, especially when compared to individuals with VI [23]. To date, FOF is typically screened by clinical interviews or self-report instruments, which mainly rely on an individual’s perception and memory and may lead to experiential bias. Thus, we propose that GI using VR technology could provide objective performance-based measures to quantify postural control due to FOF. This virtually simulated impairment in both healthy individuals and those with VI allows the examination of postural control strategies and their efficacy when transferring from standing to the first step of ambulation. Combining VR simulation with a balance measure device is a novel approach to identify balance deficits for individuals with a FOF and VI.

Therefore, the purpose of this study was to quantify the severity of FOF in people with LV compared to age- and gender-matched healthy individuals without LV. We hypothesized that individuals with LV would show a greater fear compared to healthy individuals and that the postural control strategies of people with LV during GI would be similar to those of people without LV when exposed to an immersive VR simulating FOF.

## 2. Methods

The systematic framework of this study, encompassing participant pre-task assessment, experimental design, data analysis and processing, and statistical evaluation is summarized in the proposed research pipeline.

### 2.1. Participants

**Figure 1:**
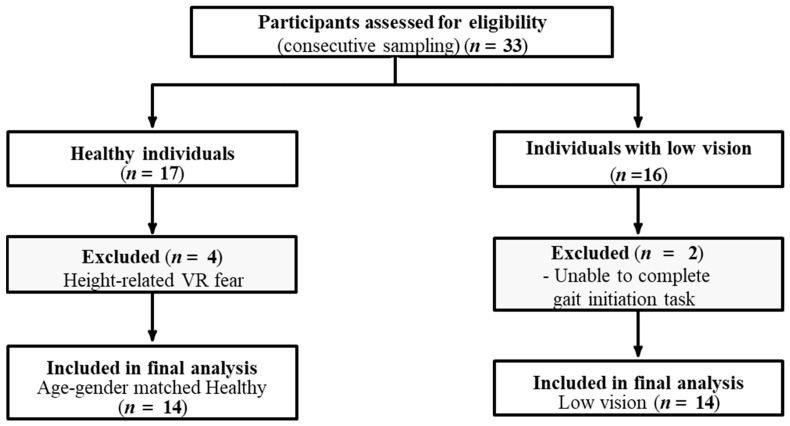
Participant flow diagram.

Fourteen adults with LV (mean age 62 ± 15 years) were recruited via consecutive sampling from UTMB Health Eye Center-Galveston through referral by Doctor of Optometry, and 14 age–gender-matched adults without LV (mean age 63 ± 10 years) were recruited from local communities. A total of 33 participants were initially assessed for eligibility; however, four healthy participants were excluded from the final analysis due to height-related VR fear, and two participants with low vision were unable to successfully complete the gait initiation task (Figure 1). Inclusion criteria for participants with LV included: being aged 18 years or older; having a diagnosis of VI confirmed through an eye examination within the last two years; reported fall within the last 24 months; capable of independent mobility, covering a minimum of 10 m without an assistive device at a comfortable speed; and able to understand the study’s procedures, consent form, and follow the study team’s instructions. Participants with a history of epilepsy, orthopedic, psychiatric, neurological, or cognitive impairments, as well as a history of substance abuse or who were currently pregnant were excluded. Low vision was defined as having a visual acuity of 20/70 or worse in the better-seeing eye or a constricted visual field of 20° or less. Ophthalmologists conducted eye examinations to ensure the credibility of participants with LV. Participants with LV represented a clinically heterogeneous group, ranging from moderate visual impairment to severe blindness, with variability in both visual acuity and visual field loss (Table 1). This heterogeneity reflects the real-world population of individuals with LV who experience balance challenges and fall risks, which may influence postural control strategies. The inclusion and exclusion criteria for participants without LV were the same except for the absence of VI, confirmed through a recent eye examination within the last two years. This study was approved by the Institutional Review Board of the University of Texas Medical Branch (UTMB IRB #21-016). All procedures were conducted in accordance with the Declaration of Helsinki, and written informed consent was obtained from all participants prior to participation.

### 2.2. Procedures

**Figure 2:**
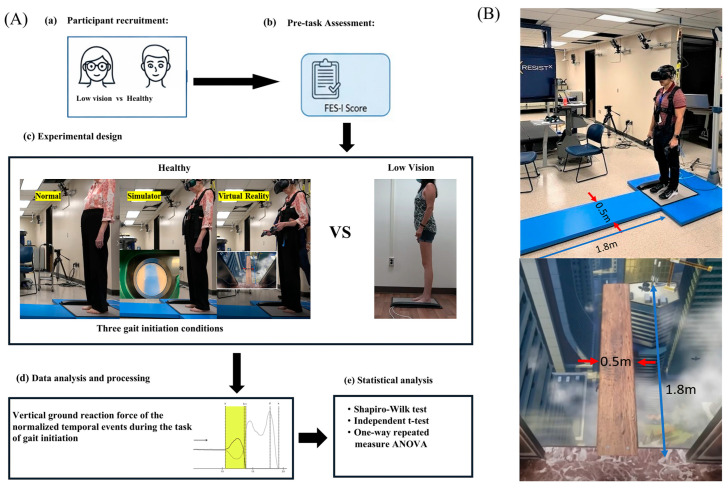
Proposed research pipeline and experimental setup. (**A**) The proposed research pipeline for evaluating the impact of visual and environmental constraints on gait initiation (GI). The framework includes: (**a**) Recruitment of healthy and low vision groups; (**b**) FOF assessment using the FES-I scale; (**c**) Experimental design comparing three GI conditions (normal, SS, and VR-induced height exposure) between groups; (**d**) Data acquisition of vertical ground reaction force using the Tekscan system; and (**e**) Statistical evaluation to verify data normality and compare group differences, illustrating the sequential research pipeline. (**B**) A healthy individual with HTC VIVE Cosmos Elite 3D VR system positioned on Tekscan High-Resolution Floor Mat for Richie’s Plank experience.

All data collection procedures were conducted by trained clinicians with experience in gait initiation and balance assessment. Prior to the task of GI, participants were assessed using the Falls Efficacy Scale International (FES-I). The 16-item FES-I was developed as a modification of the original Falls Efficacy Scale (FES) as a suitable measure to use across language and cultural contexts in the assessment of FOF [24]. While the FES assesses confidence, the FES-I measures the concern of an individual in performing a range of daily activities without falling, including activities such as transitional movements, stair negotiation, walking outside, and engaging in social activities [24]. The FES-I total score ranges from 16 to 64 with higher scores indicating greater FOF [25]. The FES-I has been found to be valid and reliable in assessing fear of falling [26,27,28] and has been used previously in individuals with visual impairment [29,30,31,32]. Following the assessment of the FES-I, both participants with and without LV walked barefoot for four meters at a self-selected comfortable pace, starting upon a verbal cue from a quiet standing position and using the Tekscan High-Resolution Floor Mat System (Tekscan Inc., South Boston, MA, USA). After each trial, participants returned to a marked starting position. Three trials were performed under this condition, with guarded assistance provided as needed. Additionally, participants without LV underwent two other conditions: GI using a non-immersive low vision sight simulator (SS; Fork in the Road, Madison, WI, USA) and GI while immersed in Richie’s Plank VR experience (see Figure 2B), specifically designed to evoke a fear of heights and falling. The VR condition was delivered using an HTC VIVE Cosmos Elite head-mounted display (HTC Corporation, Taiwan, China). The SS condition employed a non-immersive visual impairment simulator designed to reduce visual clarity and contrast, thereby mimicking low-vision conditions. The same gait initiation task instructions and verbal cues were used across all experimental conditions. Three trials were completed for each condition, with rest provided between trials as needed. All experimental procedures were completed within a single session lasting less than one hour.

### 2.3. Data Processing

**Figure 3:**
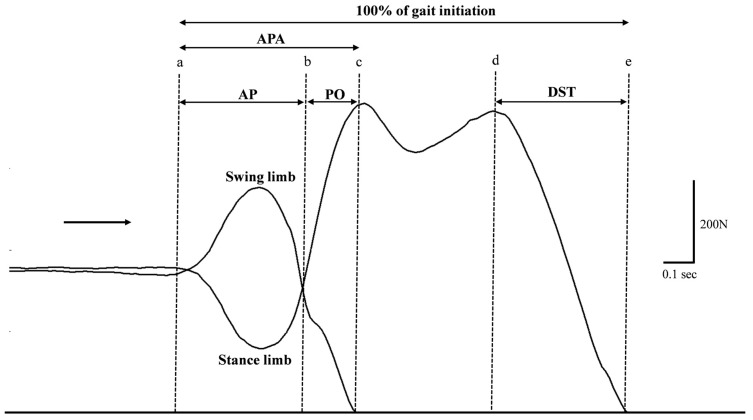
Vertical ground reaction force (Fz) of the temporal events during the task of gait initiation from one healthy individual (HK2). (a) Onset of movement, (b) Swing limb heel-off, (c) Swing limb toe-off, (d) Swing limb initial contact, and (e) Stance limb toe-off. The arrow indicates the direction of movement. Abbreviations: APA: anticipation postural adjustment phase; AP: anticipation phase; PO: push-off phase; DST: double limb support time phase.

Each temporal event of GI was captured by the vertical ground reaction force between swing and stance limbs with the Tekscan High-Resolution Floor Mat System. These temporal events of GI are illustrated in Figure 3.

To normalize temporal parameters of GI, the time from the onset of movement (Figure 3a) to stance limb toe-off (Figure 3e) was defined as 100% of the GI cycle (Figure 3). As such, relative temporal events are represented in percentages. The dependent variables are anticipation phase (AP), push-off phase (PO), anticipatory postural adjustment phase (APA), and double limb support time phase (DST) of both absolute and relative temporal events during GI. No kinematic data were collected in this study; all dependent variables were temporal events of gait initiation derived from vertical ground reaction forces.

### 2.4. Statistical Analysis

The Shapiro–Wilk test was conducted to identify the normality of all dependent variables, which include both absolute and relative temporal events as measured by vertical ground reaction forces during GI for both participants with and without LV. Additionally, Levene’s test was employed to ensure the homogeneity of variance across groups, and this assumption was satisfied (*p* > 0.05).

An independent *t*-test was employed to assess whether there was a significant difference in the levels of concern about falling between participants with and those without LV. One-way repeated measure ANOVA was used to identify changes in SS and VR compared to a normal condition during the task of GI in participants without LV regarding AP, PO, APA, and DST of both absolute and relative temporal events. Post hoc analyses were conducted using the Bonferroni post hoc test. To compare participants with and without LV, we used an independent *t*-test to assess changes in AP, PO, APA, and DST for both absolute and relative temporal events. The significance level was set at α < 0.05. All calculations were conducted using SPSS IBM version 28.

## 3. Results

### 3.1. Concerns About Falling in Individuals with LV

**Figure 4:**
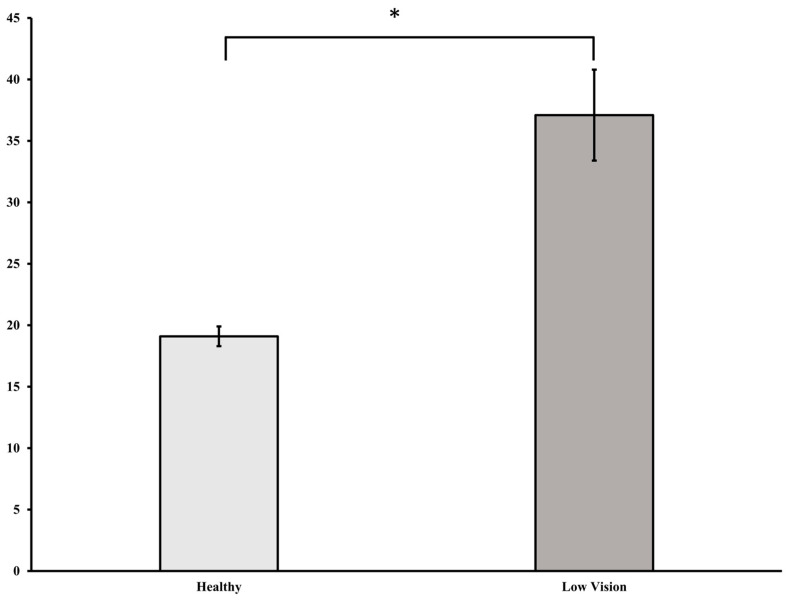
Falls Efficacy Scale International between healthy and low vision individuals. * *p* < 0.05.

For the Falls Efficacy Scale International, the independent *t*-test showed that individuals with LV had significantly greater fear compared to healthy individuals (*t* (26) = −4.789, *p* < 0.001). Please refer to Figure 4.

### 3.2. Absolute Temporal Differences in VR, SS, and Normal Conditions Among Individuals Without LV

For APA, there was no significant multivariate main effect across the VR, SS, and normal conditions, *F* (2, 12) = 2.222, *p* = 0.151. However, a significant within-subject effect was observed (with sphericity assumed), *F* (2, 26) = 4.271, *p* = 0.025. The least significant difference test revealed that the VR condition resulted in a significantly prolonged APA time compared to the normal condition during GI (*p* = 0.049).

For AP, there was no significant multivariate main effect across the normal, SS, and VR conditions, *F* (2, 12) = 1.784, *p* = 0.210. For PO, a significant multivariate main effect was observed across the normal, SS, and VR conditions, *F* (2, 12) = 8.424, *p* = 0.005. However, no significant within-subject effects were detected (with sphericity assumed), *F* (2, 26) = 1.650, *p* = 0.212. The least significant difference test showed that the VR condition resulted in a significantly prolonged PO time compared to the normal condition during GI (*p* = 0.033). For the GI cycle, there was no significant multivariate main effect across the normal, SS, and VR conditions, *F* (2, 12) = 1.627, *p* = 0.237. Additionally, there were no significant within-subject effects (sphericity assumed), *F* (2, 26) = 2.815, *p* = 0.078. However, the least significant difference test revealed that the VR condition led to a significantly prolonged GI cycle compared to the normal condition during GI (*p* = 0.049).

For DST, data did not meet normality assumptions; therefore, this study employed a Friedman test. This test indicated significant differences across the normal, SS, and VR conditions during GI in individuals without LV, *χ^2^*(2, *N* = 14) = 6.157, *p* = 0.046. Subsequent post hoc analyses showed that the VR condition (mean = 0.511 ± std = 0.718) had a significantly prolonged time compared to the SS condition (mean = 0.262 ± std = 0.165) (*p* = 0.018).

### 3.3. Comparative Analysis of Absolute Temporal Events Between LV and Individuals Without LV

**Figure 5:**
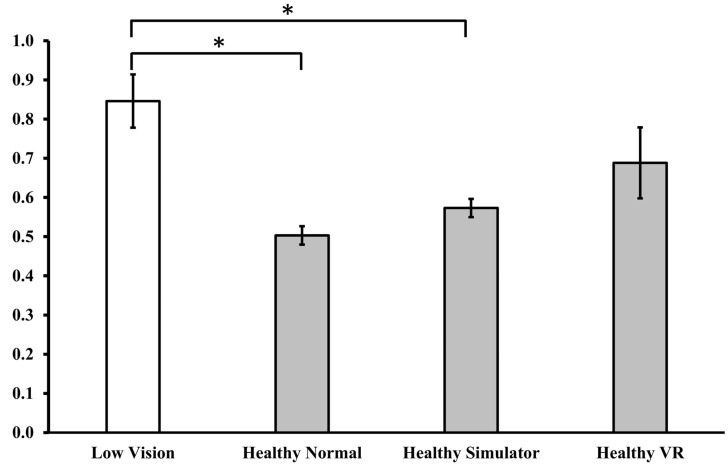
Anticipatory postural adjustment duration in absolute time between LV and healthy individuals. * *p* < 0.05. Abbreviations: VR (virtual reality).

For APA, the independent *t*-test revealed that individuals with LV exhibited a significantly prolonged APA duration compared to both the normal (*p* < 0.001) and SS (*p* < 0.001) conditions. However, the APA duration in the LV group did not differ from the VR condition (*p* = 0.320). Please refer to Figure 5. The independent *t*-test also showed that individuals with LV had a significantly prolonged AP time compared to the normal (*p* < 0.001), SS (*p* < 0.001), and VR (*p* = 0.049) conditions. For PO, the independent *t*-test indicated no significant differences between individuals with LV and the normal (*p* = 0.272), SS (*p* = 0.780), or VR (*p* = 0.601) conditions. Regarding the GI cycle’s duration, the independent *t*-test found that individuals with LV had a significantly prolonged duration compared to both the normal (*p* < 0.001) and SS (*p* < 0.006) conditions, but this duration was not different from the VR condition (*p* = 0.830). For DST, since the data did not meet the assumption of normality, a Mann–Whitney U test was used. The test revealed that individuals with LV did not significantly differ from individuals without LV in the normal (*U* = 59.5, *p* = 0.077), SS (*U* = 59.5, *p* = 0.077), or VR (*U* = 100.5, *p* = 0.910) conditions.

### 3.4. Relative Temporal Differences in VR, SS, and Normal Conditions Among Individuals Without LV

For AP, there was no significant multivariate main effect across the normal, SS, and VR conditions, *F* (2, 12) = 0.14, *p* = 0.986. Similarly, no significant multivariate main effects were observed for PO and APA across the normal, SS, and VR conditions, with *F* (2, 12) = 1.571, *p* = 0.248, and *F* (2, 12) = 0.234, *p* = 0.795, respectively. For DST, since the data did not meet the assumption of normality, this study utilized a Friedman test. The test revealed no significant differences across the normal, SS, and VR conditions during GI in individuals without LV, *χ^2^*(2, *N* = 14) = 3, *p* = 0.223.

### 3.5. Comparative Analysis of Relative Temporal Events Between LV and Healthy Individuals

For APA, the independent *t*-test revealed that individuals with LV had a significantly prolonged APA duration compared to VR (*p* = 0.004), SS (*p* < 0.001), and normal (*p* < 0.001) conditions. Similarly, the independent *t*-test showed that individuals with LV had a significantly prolonged AP time when compared to the normal (*p* < 0.001), SS (*p* < 0.001), and VR (*p* < 0.001) conditions. However, for PO, the independent *t*-test indicated that there were no significant differences between individuals with LV and the VR (*p* = 0.746), SS (*p* = 0.316), and normal (*p* = 0.399) conditions.

For DST, as the data did not satisfy the assumption of normality, this study employed the Mann–Whitney U test. The results indicated that individuals with LV did not significantly differ from individuals without LV in the normal (*U* = 127, *p* = 0.194), SS (*U* = 101, *p* = 0.910), or VR (*U* = 123, *p* = 0.265) conditions.

## 4. Discussion

This study investigated the severity of fear-related changes in postural control in people with LV compared to age–gender-matched individuals without LV. The findings provide compelling evidence that individuals with LV demonstrate distinct postural control strategies that differ from individuals without LV and even from those individuals without LV in a fear-evoking VR environment. These unique strategies observed in individuals with LV reflect the unique challenges and the adaptations adopted by those with LV. This capacity of individuals with LV to adapt and adjust their postural control strategies in response to task and environmental demands has broader implications for fall prevention strategies and rehabilitation programs.

When assessing absolute temporal changes in the VR, SS, and normal conditions among individuals without LV, the AP remained unchanged, which is in contrast with the study’s findings of significantly prolonged PO in the VR environment. The unchanged AP among participants without LV underlies the stability of postural control even during challenging conditions. However, the finding of prolonged PO emphasizes that even in the absence of visual impairments, fear-evoking stimuli such as fear of heights and falling can create hesitations and alterations in postural control strategies. This finding suggests that the significantly prolonged APA duration could be attributed to an increased duration of PO in response to a fear-evoking stimulus during the task of GI for individuals without LV. Additionally, the study’s findings illustrated that the participants with LV demonstrated movement hesitations or adjustments comparable to participants without LV in the VR environment. Distinctly, however, participants with LV exhibit significantly longer AP duration compared to all conditions of participants without LV.

**Figure 6:**
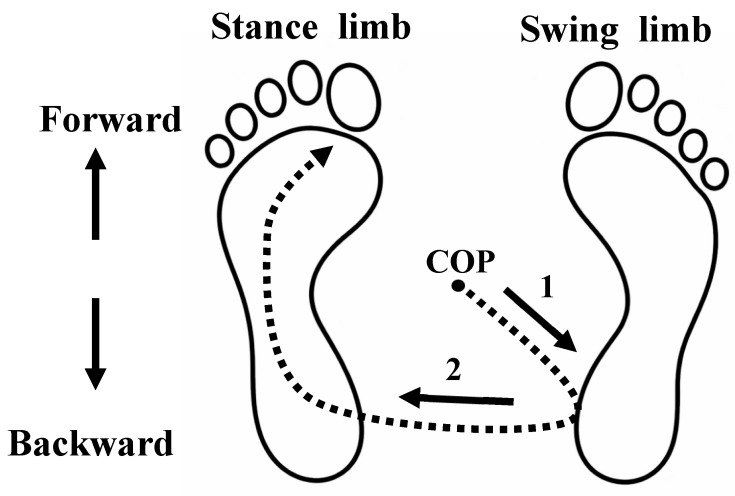
Dynamic weight shifting between the swing and stance limbs during gait initiation. 1. Backward center-of-pressure (COP) shift with weight transfer toward the swing limb (loading) and concurrent unloading of the stance limb. 2. Subsequent weight transfer toward the stance limb (loading) accompanied by unloading of the swing limb.

**Figure 7:**
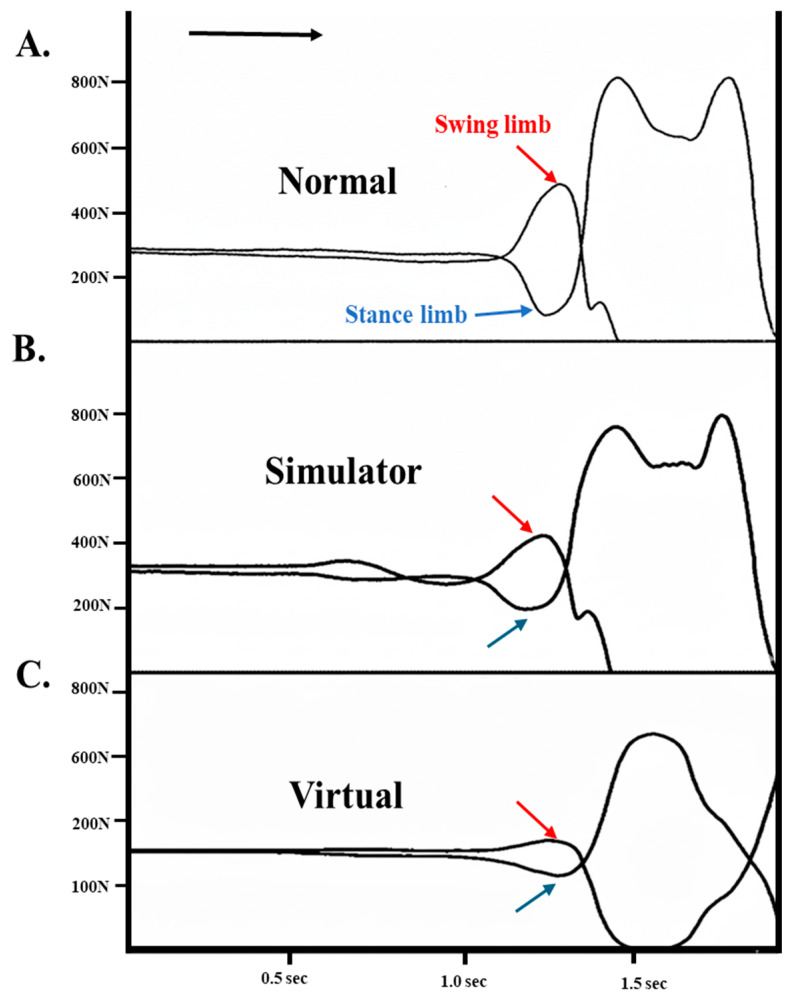
Vertical ground reaction forces of the stance and swing limbs during gait initiation under visually challenging conditions in healthy individuals. The figure illustrates a progressive reduction in stance limb unloading and swing limb loading during anticipatory postural adjustments across (**A**) normal, (**B**) SS, and (**C**) VR conditions.

GI represents a critical transitional phase between quiet standing and dynamic walking, during which a stable postural equilibrium is intentionally disrupted. This transition is primarily governed by a feedforward motor program rather than reactive feedback-based control. As previously established, GI is mediated by APAs, characterized by reciprocal inhibition of the stance limb plantar flexors and activation of the dorsiflexors [33,34,35,36]. These coordinated muscle actions generate a posterior and lateral shift in the COP toward the swing limb, followed by a rapid forward transfer toward the stance limb to facilitate the transition from a static to a dynamic state (please see Figure 6). Because this process necessitates deliberate destabilization of balance, it may evoke fear and hesitation, particularly in individuals with low vision. Importantly, such hesitation is unlikely to reflect delayed sensory feedback processing; rather, it appears to manifest as a conservative modulation of the feedforward motor program. The present findings demonstrate a progressive reduction in stance limb unloading and swing limb loading under visually challenging conditions, indicating diminished weight shifting during APAs (please see Figure 7).

Similar attenuations in COP excursion have been reported in populations with impaired balance control, such as frail older adults and individuals with hemiparesis, who adopt conservative anticipatory strategies to prioritize stability over mobility [33,36]. Notably, the movement strategies observed in individuals with low vision were comparable to those seen under fear-inducing virtual height conditions, characterized by constrained dynamic weight shifting. This quantitative reduction in anticipatory weight transfer likely reflects a chronic adaptive response to fear of falling, highlighting the severity and persistence of balance-related anxiety in the low-vision population.

These findings were reflected in the relative temporal events, in which people with LV exhibited a significantly prolonged APA duration, primarily due to an extended AP time compared to all conditions (VR, SS, and normal) observed in participants without LV. This finding further suggests that individuals with LV might experience even greater temporal hesitations than individuals without LV in fear-evoking virtual simulations during GI, suggesting that participants with LV exhibit greater FOF than those without LV. Concordantly, the participants in this study with LV reported greater concerns with falling on the FES-I compared to those without LV, agreeing with findings of previous studies assessing self-reported concerns with falling [32]. Interestingly, while participants without LV showed an extended PO in the VR condition, individuals with LV exhibited a longer AP duration, suggesting that individuals with LV employ distinct postural strategies to adapt to their fear before executing the first step from a static standing position (GI). However, most of the work previously has explored GI in other populations or has explored overall postural control, balance, or gait stability in individuals with LV, while limited work exists assessing the characteristics of GI in people with LV despite the known relationship of prolonged GI to multiple falls. GI has been strongly associated with vision and older adults demonstrate a delayed sensory reweighting process to compensate for VI [11]. Individuals diagnosed with VI demonstrate postural control mechanisms different from those of individuals without LV, which are further compensated by the remaining intact sensory systems [9,21,22,37,38,39,40]. If a delay in the sensory reweighting process exists in individuals with LV, individuals with LV may demonstrate prolonged APAs during GI.

Taken together, the present findings on GI in individuals with LV should be interpreted in light of the distinction between functional capacity and real-world performance. As these data were collected in a controlled laboratory environment, they primarily reflect participants’ capacity under safe and predictable conditions. In daily community life, individuals with LV are exposed to additional contextual factors such as uneven surfaces, environmental obstacles, variable lighting, time constraints, and cognitive demands, which may further shape fear-related postural control strategies. Thus, while providing an important foundation for understanding the postural strategies during GI, these findings suggest the need for future research to examine how such strategies manifest in real-world settings. In addition, although kinematic data were not extracted in the present study, future investigations may incorporate kinematic analyses to further characterize movement strategies and fall-related mechanisms during APAs.

### 4.1. Clinical Implications

This present study’s findings draw attention to the unique postural control strategies adopted by individuals with LV, suggesting the need for a tailored approach in low vision rehabilitation programs [41]. Prior research has shown that despite professional recommendations for preferred practice patterns proposed by the American Academy of Ophthalmology in 2017 [42], barriers to low vision rehabilitation hinder the utilization of low vision rehabilitation in regular ophthalmology practice [39]. Given the evident delays in APA during GI in these individuals, targeted interventions aiming to improve postural control during a steady state walking and GI hold the potential to reduce the risks associated with FOF and falls.

Prior studies in LV interventions show the benefits of LV devices or aids, such as electronic magnifiers, electronic walking sticks, and smart glasses, which could act as compensatory strategies to address the functional needs of individuals with LV [43,44,45]. However, low vision rehabilitation needs to be multidisciplinary to address the multidimensional factors related to FOF in individuals with LV, especially since the provision of visual aids only for these individuals is ineffective in changing their FOF [46]. A recent systematic review in people with neurological disorders showed that a combination of gait training with lower limb training and balance exercises were effective in reducing FOF in individuals with Parkinson’s disease, while individuals with multiple sclerosis also benefitted from the addition of home-based and leisure exercise in FOF reduction [47]. These principles extend naturally to stroke survivors, in whom FOF frequently exacerbates impaired GI. Inefficient APAs and inadequate limb loading not only hinder mechanical propulsion but also act as psychological barriers, reinforcing fear-related mobility restrictions. Consequently, stroke rehabilitation must evolve beyond static training to incorporate dynamic, task-specific interventions. By actively targeting APAs through controlled weight-shifting tasks and strategic GI protocols, clinicians may enhance the functional loading of the paretic limb [33]. Addressing these biomechanical foundations may therefore provide a dual benefit by improving physical stability while simultaneously reducing FOF that limits functional recovery. In addition to addressing the motor and physical aspects of FOF, the addition of cognitive behavioral therapy holds promise in reducing FOF and improving balance performance in older individuals with FOF [48]. These unique findings amongst different populations in which FOF is prevalent, however, underscore the need for more rigorous studies that explore specific low vision rehabilitation interventions that could positively influence FOF and subsequent fall risk in individuals with LV.

In addition to participating in physical exercise and strength training [42,49], more recent studies have explored the potential of training sensory reweighting and its potential role in addressing maladaptive postural control strategies in individuals with LV. Sensory reweighting programs may be integrated into standard low vision rehabilitation programs to help train the brain to more efficiently utilize intact sensory systems in the absence of adequate visual input. Specifically, given that the vestibular system is crucial for postural control, vestibular exercise programs may hold potential benefits for individuals with LV. After a 3-month vestibular stimulating exercise regime, the 28 individuals with visual impairments showed postural stability comparable to the 42 individuals without visual impairments in stabilometry tests with eyes closed [50]. LV rehabilitation emphasizes the importance of integrating sensory reweighting and vestibular exercise programs to enhance postural control, particularly as balance and vestibular exercises aim to improve the sensory reweighting process and may be beneficial for mitigating prolonged APAs in individuals with LV; however, further studies are needed to assess their effect on GI specifically.

### 4.2. Limitations

In addition to the small sample size, our study focused primarily on fear-evoking environments (fear of heights) in a controlled VR-simulated setting. Real-world scenarios pose different challenges and stimuli that were not replicated in this study. Future studies should explore the implications of these findings in larger samples and more varied and unpredictable settings to better understand the dynamic interaction of postural control, FOF, VI, and environment and task demands in individuals with LV. Although amplitude was not treated as a dependent variable in the present study, incorporating normalized amplitude measures may help address inter-subject variability and enhance the comparability of gait-related outcomes across participants.

## 5. Conclusions

This study demonstrates that individuals with LV who self-report a FOF exhibit objectively impaired GI, characterized by significantly prolonged APAs. Our findings reveal that while fear-evoking environments cause temporary hesitations in sighted individuals, those with LV adopt a chronically conservative feedforward motor program, specifically lengthening the initial phase of postural preparation, referred to as the anticipation phase. Since vision plays a pivotal role in how we prepare and initiate movement, acting as one of the primary senses we use to safely navigate our environment, its impairment can markedly alter how individuals anticipate and interact with their physical environment during movement transitions that require deliberate destabilization of static balance, such as gait initiation. Consequently, these results suggest that proactive clinical referrals and multidisciplinary rehabilitation strategies incorporating sensory reweighting and vestibular training are essential to effectively address FOF, optimize postural control, and mitigate fall risks in individuals with LV.

## Figures and Tables

**Table 1:** Demographic information of participants with low vision.

Low Vision Subject	Gender	Age	Height (cm)	Weight (kg)	BMI	VA OD	VA OS	Visual Impairment Classification	FES-I
1	Female	50	163	62	24	20/400	20/125	Moderate Visual Impairment	34
2	Male	45	192	117	32	20/400	20/400	Blindness	45
3	Female	79	160	73	29	CF at 3ft	CF at 3ft	Severe Blindness	44
4	Male	70	178	115	36	20/150	20/150	Moderate Visual Impairment	22
5	Female	67	152	76	33	20/300	20/300	Blindness	46
6	Female	66	168	83	29	20/200	20/400	Moderate Visual Impairment	19
7	Female	70	173	100	34	20/400	CF at 3ft	Blindness	39
8	Male	59	168	83	30	20/400	NLP	Blindness	36
9	Male	87	157	76	31	20/400	CF at 1ft	Blindness	44
10	Female	68	152	62	27	20/400	20/100	Moderate Visual Impairment	55
11	Male	26	173	80	27	CF at 3ft	CF at 3ft	Severe Blindness	23
12	Male	75	170	72	25	20/200	20/200	Moderate Visual Impairment	18
13	Male	50	168	80	29	20/80	CF at 3ft	Moderate Visual Impairment	31
14	Female	55	168	97	34	20/70 * Constricted VF 10 degrees	20/20	Severe blindness	64
Mean ± SD		62 ± 15	167 ± 11	84 ± 17	30 ± 4				37 ± 14

* Severe blindness was classified based on a constricted visual field of 10 degrees rather than visual acuity. Abbreviations: VA: Visual acuity, OD: Oculus dexter (right eye), OS: Oculus sinister (left eye), CF: Count Finger, VF: Visual field, FES-I: Falls Efficacy Scale International.

## Data Availability

The data presented in this study include de-identified human participant data derived from clinical electronic medical records (EPIC) and balance assessments. Due to ethical, legal, and privacy restrictions, the data are not publicly available. Data are available from the corresponding author upon reasonable request and subject to institutional and IRB regulations.
